# Copulatory mechanics of ghost spiders reveals a new self‐bracing mechanism in entelegyne spiders

**DOI:** 10.1002/ece3.10582

**Published:** 2023-10-03

**Authors:** Dante Poy, Luis N. Piacentini, Shou‐Wang Lin, Leonel A. Martínez, Martín J. Ramírez, Peter Michalik

**Affiliations:** ^1^ Division of Arachnology Museo Argentino de Ciencias Naturales – CONICET Buenos Aires Argentina; ^2^ Zoologisches Institut und Museum Universität Greifswald Greifswald Germany

**Keywords:** Anyphaenidae, cryofixation, genitalia, micro‐CT, sexual selection, sperm transfer

## Abstract

Spiders evolved a distinctive sperm transfer system, with the male copulatory organs located on the tarsus of the pedipalps. In entelegyne spiders, these organs are usually very complex and consist of various sclerites that not only allow the transfer of the sperm themselves but also provide a mechanical interlock between the male and female genitalia. This interlocking can also involve elements that are not part of the copulatory organ such as the retrolateral tibial apophysis (RTA)—a characteristic of the most diverse group of spiders (RTA clade). The RTA is frequently used for primary locking i.e., the first mechanical engagement between male and female genitalia. Despite its functional importance, some diverse spider lineages have lost the RTA, but evolved an apophysis on the femur instead. It can be hypothesized that this femoral apophysis is a functional surrogate of the RTA during primary locking or possibly serves another function, such as self‐bracing, which involves mechanical interaction between male genital structures themselves to stabilize the inserted pedipalp. We tested these hypotheses using ghost spiders of the genus *Josa* (Anyphaenidae). Our micro‐computed tomography data of cryofixed mating pairs show that the primary locking occurs through elements of the copulatory organ itself and that the femoral apophysis does not contact the female genitalia, but hooks to a projection of the copulatory bulb, representing a newly documented self‐bracing mechanism for entelegyne spiders. Additionally, we show that the femoral self‐bracing apophysis is rather uniform within the genus *Josa.* This is in contrast to the male genital structures that interact with the female, indicating that the male genital structures of *Josa* are subject to different selective regimes.

## Graphical abstract

The genitalia of spiders are known for their complex interactions during mating. In addition to the elements of the copulatory organs themselves, other structures may be involved in the copulatory process. Here, we reveal the function of a conspicuous femoral apophysis that demonstrates a new self‐bracing mechanism for entelegyne spiders.

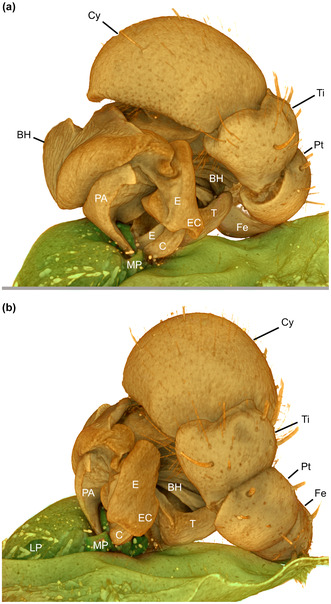

## 1 INTRODUCTION

Copulatory organs have originated many times among animals as efficient structures for transferring sperm directly to the female. Beyond this fundamental function, the strong selective pressures on copulatory structures, particularly sexual selection, have led to an enormous diversity of forms and functions (Leonard & Córdoba‐Aguilar, 2010). Much of our knowledge on the evolutionary dynamics of genital organs relies on detailed knowledge of the mechanical interactions that occur during mating, which may be exceptionally intricate in arthropods (Simmons, 2014). Spiders have a distinctive sperm transfer system, with the male copulatory organs (i.e., copulatory bulbs) located on the tarsus (i.e., cymbium) of the pedipalps lacking a direct connection with the testes. Males must therefore charge both of their sperm reservoirs (i.e., spermophor) located in the copulatory bulbs with sperm before copulation—a process known as sperm induction (see Foelix, 2011). The sperm is then transferred to the females by inserting the intromittent sclerite (i.e., embolus) into the female copulatory opening (i.e., pedipalpal insertion).

The reproductive system of spiders can be classified into two main types based on the organization of the female genitalia: haplogyne and entelegyne (see Eberhard & Huber, 2010). In contrast to the haplogyne type, characterized by sperm entering and exiting the sperm storage organ via the same duct, in the entelegyne type, females have a pair of specialized copulatory openings located in an external, rigid genital plate (i.e., epigyne) in front of the genital opening. These copulatory openings lead to the copulatory ducts, which disembogue into the sperm storage organs (i.e., spermathecae). Sperm exits the spermathecae by a separate set of ducts (i.e., fertilization ducts) connecting the spermathecae with the uterus externus (see Uhl et al., 2010: fig. 2). Entelegyne male genitalia lack muscles and are usually composed of many structures connected by inflatable membranes (i.e., hematodochae) resulting in a complex appearance (Eberhard & Huber, 2010; Huber, 2004). During genital coupling, entelegyne male genitalia depend on the hydraulic expansion of the hematodochae, which causes a sequential locking of the male genital structures into corresponding structures of the epigyne (Eberhard & Huber, 1998, 2010).

Since many mechanical interactions may occur during the genital coupling of entelegyne taxa, at least four different types of genital mechanisms can be recognized, in the following order (see, e.g., Poy et al., 2023). The first type is the primary locking, which is the initial—and usually the strongest—mechanical engagement between male and female genitalia, prior to full hematodochal expansion. The second type are the secondary lockings, which occur after primary locking, with full hematodochal expansion, and serve to further lock male and female genitalia in functional contact. The third type is the functional conduction, which is the guidance of the male embolus through one of the female copulatory openings, usually accomplished by a furrowed male genital structure. Finally, the fourth type is the self‐bracing, which encompasses any mechanical interaction between male genital structures themselves to facilitate structural stability of the inserted pedipalp.

The RTA clade, which is the most diverse group of entelegyne spiders, and of spiders as a whole as well, is characterized by complex genital mechanics that involve many elements of the copulatory bulb (see Huber, 1995a; Poy et al., 2023; and references therein included). The primary locking is usually achieved by the characteristic retrolateral tibial apophysis (RTA) of the male pedipalp (Huber, 1995a; Poy et al., 2023), which usually fits in some female genital cavity (e.g., as in *Anyphaena accentuata*, see Huber, 1995b) and is the name‐giving synapomorphy for this clade (Coddington & Levi, 1991). The RTA therefore holds a crucial role in the genital coupling, hence it is puzzling that, despite its functional relevance, it was repeatedly lost or reduced in several clades, as for example in Lycosidae (see Piacentini & Ramírez, 2019), many Castanieirinae (Corinnidae) (see Reiskind, 1969) and many *Lyssomanes* species (Salticidae) (see Galiano, 1980). The few experimental studies on genitalia mechanics of RTA clade species lacking the RTA showed that the primary locking is carried out only by elements of the copulatory bulb. For example, in Lycosidae, primary locking is achieved by the locking of the median apophysis in a female anterior epigynal pocket (*Lycosa chaperi*, *L. wroughtoni* and *Pardosa amentata*; Osterloh, 1922; Sadana, 1972, 1981), or by locking the palea in a lateral epigynal pocket (*Agalenocosa pirity*; Poy et al., 2020).

Numerous representatives of Amaurobiodinae (Anyphaenidae) have also undergone losses or reductions of the RTA (see Ramírez, 2003), of which the genus *Josa* is an example. This Neotropical genus is highly speciose and can be distinguished from other amaurobioidines by the males having a hook‐like femoral apophysis (Ramírez, 2003). The function of this apophysis is unknown, but it can be hypothesized (1) to act as a novel functional replacement of the missing RTA used for primary locking into the female genitalia or (2) being involved in self‐bracing. In this study, we test these hypotheses by conducting a thorough analysis of the genitalic interaction of two species of *Josa* (*J. calilegua* and an undescribed species) using micro‐computed tomography (micro‐CT) of cryofixed mating pairs, and provide a detailed account of female and male genitalia using optical and scanning electron microscopy.

## 2 MATERIALS AND METHODS

### 2.1 Collection and rearing of specimens

Juvenile specimens of *Josa calilegua* Ramírez, 2003 and of an undescribed closely related species (*Josa* sp.) were collected in November 2021 in Calilegua National Park, Jujuy, Argentina. After collection, the juvenile specimens were taken to the Museo Argentino de Ciencias Naturales (MACN) and reared to maturity. During this period the spiders were kept in plastic Petri dishes under controlled conditions (room temperature 25°C, water supplied by a moistened cotton) and fed twice a week with fruit flies (*Drosophila melanogaster*) or houseflies (*Musca domestica*) depending on their size.

### 2.2 Cryofixation of mating pairs

For the cryofixation of mating pairs, a virgin adult female was placed and maintained individually in a plastic tube of 4 cm long and 1 cm in diameter. The base of the tube was previously sectioned, and a cotton plug was placed instead. This was done to allow flowing of liquid nitrogen through the tube, as any accumulation of liquid nitrogen during cryofixation can lead to the destruction of the spiders (liquid nitrogen boils violently once poured into the tube). After an acclimatization period of 30 min, a virgin adult male was introduced into the tube. If the spiders engaged in copulation, the tube was carefully placed vertically into a holder and the lid was removed and replaced by a funnel. A small amount of liquid nitrogen (−195.8°C) was then poured into the funnel at the moment a pedipalp insertion occurred. If copulation did not occur within an hour, the pair was recovered from the tube. Successive trials were made after washing the tube with 96% ethanol. After cryofixation, the pair was transferred immediately to cold 80% ethanol (−18°C) and stored at −18°C for 3 weeks. After this period, the spiders were dehydrated in a graded series of ethanol (96%–100%), exchanging the alcohol for a higher grade every one week (as in Poy et al., 2020).

### 2.3 Micro‐CT and morphological analyses

The cryofixed mating pairs were examined with X‐ray microscopy. For this, the already dehydrated pairs were stained in a 1% iodine solution for 12 h and then washed in 100% ethanol (as in Poy et al., 2020). Subsequently, the sample was dried in hexamethyldisilazane (Thermo Fisher Scientific Inc.) and then scanned with an Xradia MicroXCT‐200 X‐ray imaging system (Carl Zeiss Microscopy GmbH) using the 4× and 10× lenses. Tomographic projections were reconstructed using the XMReconstructor software (Carl Zeiss Microscopy GmbH). The segmentation of genital structures and the virtual reconstructions of the image stack were performed using Amira 6.4 (Visualization Science Group, FEI). Furthermore, Drishti 3.1 (National Computational Infrastructure's VizLab) was used for volume reconstruction of the coupled genitalia. For the construction of the interactive PDF models, the segmented structures were converted to a surface mesh by Fiji (Schindelin et al., 2012). These files were subsequently imported into MeVisLab (MeVis Medical Solutions AG and Fraunhofer MEVIS) using the “Scientific3DFigurePDFApp” module, reduced, colored, and exported as u3D files, which were then inserted into the additional files in the pdf format (Adobe Acrobat Pro). Digital images of the genitalia of preserved specimens of both *Josa* species were taken with a Leica M165 C stereomicroscope equipped with a Leica DFC 290 digital camera. Extended focal range images were composed with Helicon Focus 4.62 (Helicon Soft Ltd.). For morphological description, the female and male genitalia of *Josa* sp. were dissected and visualized using scanning electron microscopy (SEM). The SEM samples were dehydrated with a graded series of ethanol (80%, 96%, and 100%), then critical point dried and sputter‐coated with gold–palladium. Images were taken in high vacuum in a FEI XL30 TMP (Thermo Fisher Scientific Inc.). The voucher numbers of the cryofixed specimens used for these analyses are included in the corresponding figure legends.

## 3 RESULTS

### 3.1 Genital morphology

The genital morphology in *Josa* is very diverse, however, the general organization and the functioning of the genital structures is nearly identical in both of the species studied. Thus, although the results are mainly described based on data from *Josa* sp., the description and interpretation of the data can be generalized for both species.

**FIGURE 1:**
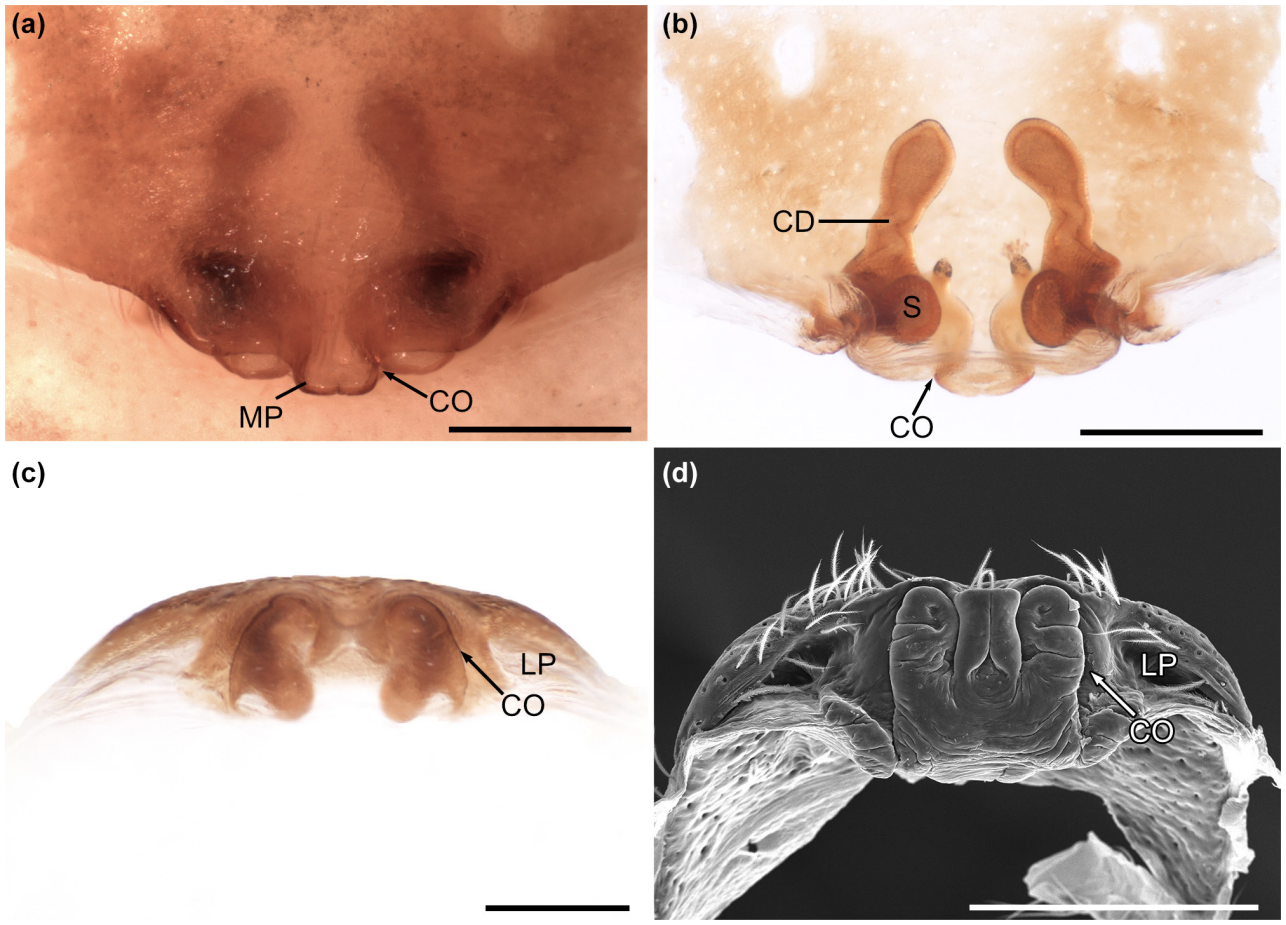
*Josa* sp. female genitalia. Dissected epigyne: (a) ventral view; (b) dorsal view; (c) posterior view. Dissected epigyne, SEM image: (d) posterior view. Scales: (a–d) 200 μm. CD, copulatory duct; CO, copulatory opening; LP, lateral pocket; MP, median pocket; S, spermatheca.

The epigyne of *Josa* sp. is a sclerotized plate, which has a characteristic median pocket (Figure 1a). The wide copulatory ducts are convoluted, ending in two globular spermathecae (Figure 1b). From a posterior view, two copulatory openings and two slightly sclerotized lateral pockets are evident (Figure 1c,d).

**FIGURE 2:**
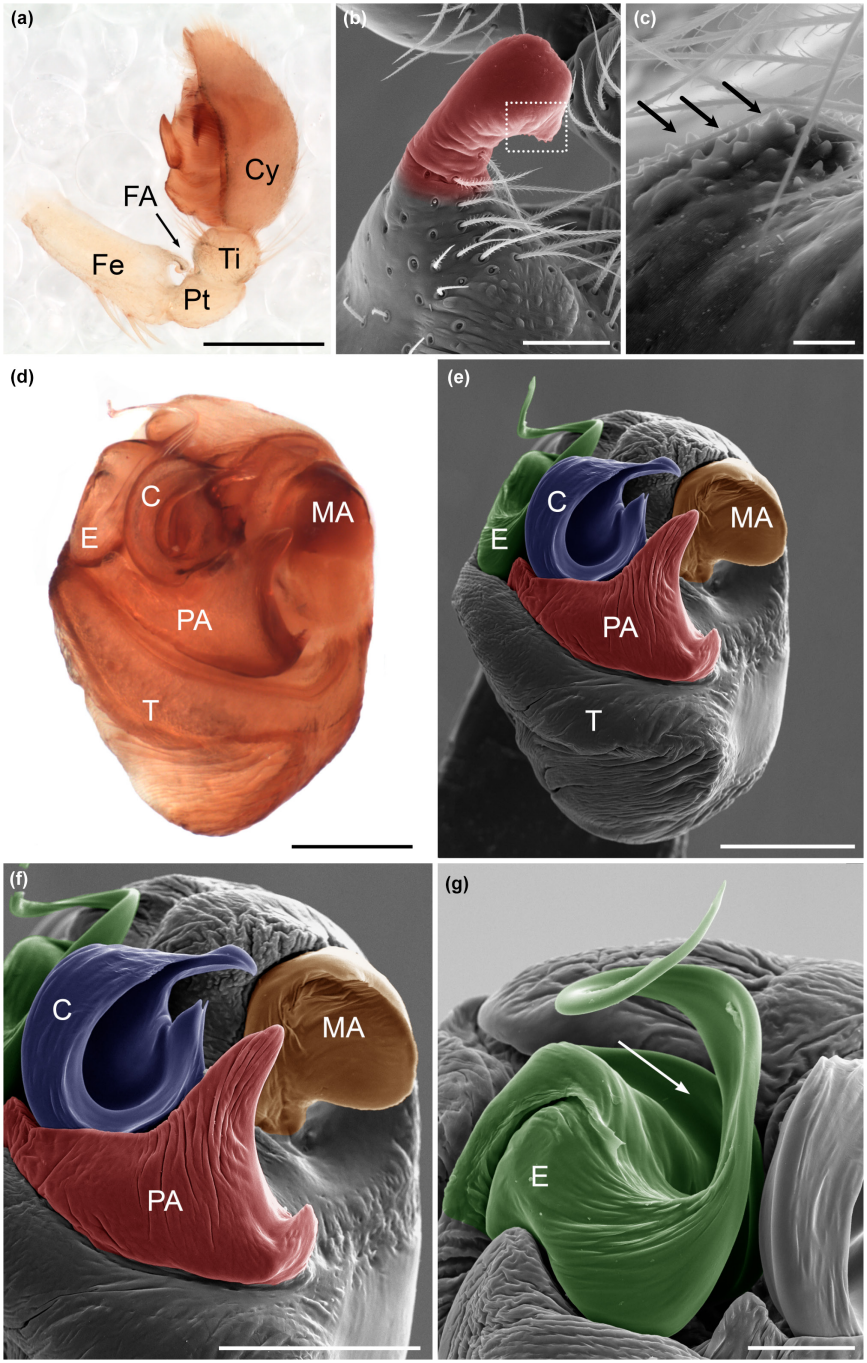
*Josa* sp. Male genitalia. Dissected left pedipalp: (a) retrolateral view. (b, c) SEM images of femoral apophysis (highlighted in red) showing the denticles in its distal part (arrows). Dissected left copulatory bulb: (d) ventral view. Dissected left copulatory bulb, SEM images: (e) ventral view; (f), ventral view, detail of sclerites; (g) prolateral view, embolus detail. Scales: (a) 500 μm; (b, g) 50 μm; (c) 10 μm; (d) 200 μm; (e, f) 100 μm. The white arrow indicates the basal EC. C, conductor; Cy, cymbium; E, embolus; Fe, femur; FA, femoral apophysis; MA, median apophysis; PA, paramedian apophysis; Pt, patella; T, tegulum; Ti, tibia.

The male pedipalp of *Josa* sp. has distinctive characteristics. The femur is relatively long, with a ventral hook‐shaped apophysis in the distal end, and the tibia and patella are short (Figure 2a). The tip of the femoral apophysis has many denticles (Figure 2b,c). On the copulatory bulb, from a ventral view, all of the characteristic sclerites of Amaurobioidinae (Ramírez, 2003) can be distinguished, i.e., the conductor, a median apophysis, a paramedian apophysis, and the embolus (Figure 2e,f). The conductor is semicircular, with an internal furrow and a hook‐like tip (Figure 2f). The median apophysis is placed on the retrolateral side, spoon‐shaped, very wide and forming a crest (Figure 2f). The paramedian apophysis is well‐developed, triangular, with a stout base that rises into a spike (Figure 2f). The embolus is mostly lamellar and has a crest on its base (basal embolar crest [EC]) that can be seen prolaterally (Figure 2g).

**FIGURE 3:**
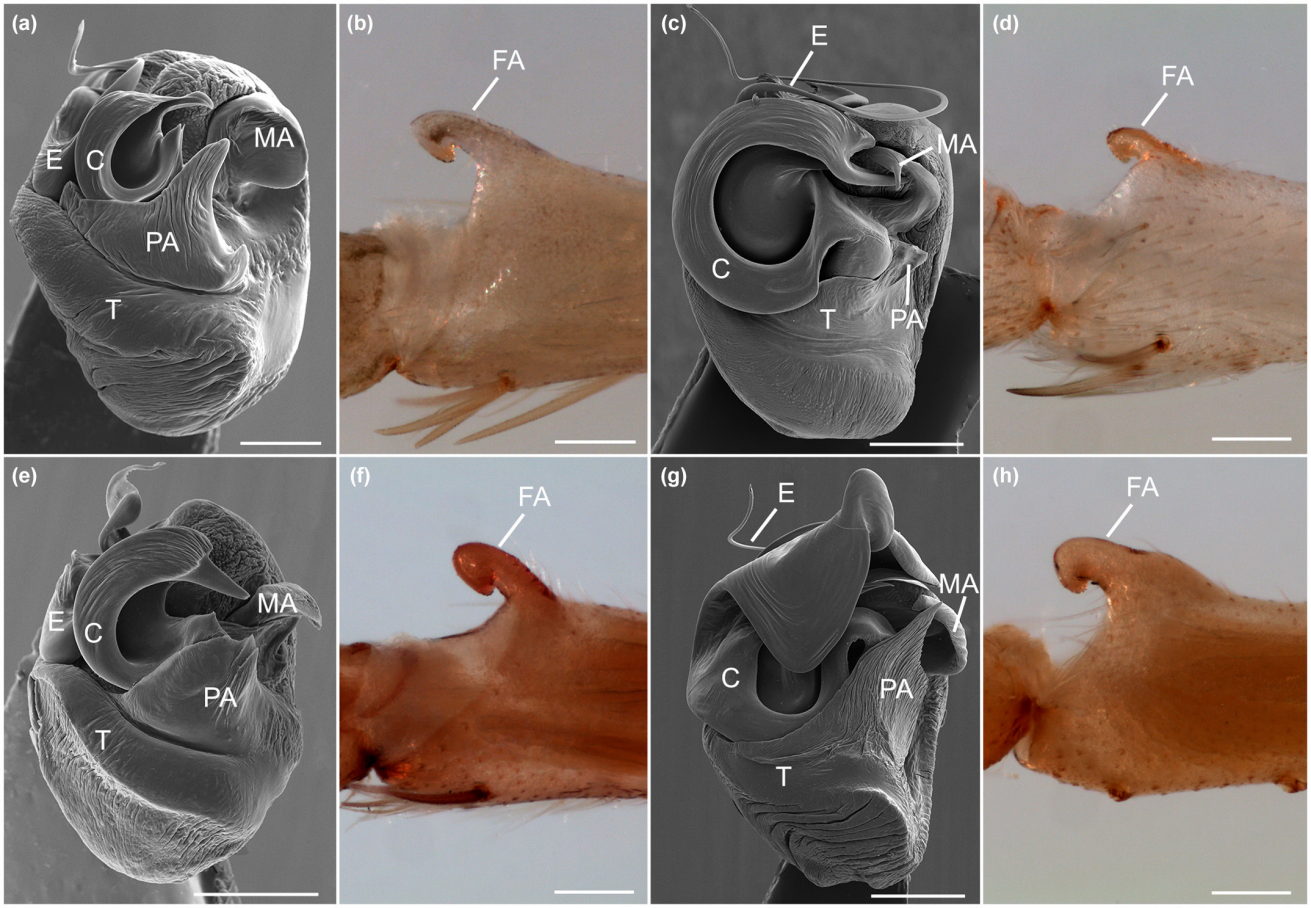
Variation in the male genital morphology in species of the genus *Josa*. Dissected left copulatory bulbs in ventral view, SEM images (a, c, e, g), and detail of the femur showing femoral apophysis (b, d, f, h). (a, b) *Josa* sp., (c, d) *Josa calilegua*, (e, f) *Josa andesiana*, (g, h) *Josa nigrifrons*. Note that the variation in the shape and size of the copulatory bulb structures that contact the female (E, C, MA, and PA) is greater than the variation in the femoral apophysis. Scales: (a) 100 μm; (b–h) 200 μm. C, conductor; E, embolus; FA, femoral apophysis; MA, median apophysis; PA, paramedian apophysis; T, tegulum.

A morphological comparison to *J. calilegua* and two additional *Josa* species (*J. nigrifrons* [Simon, 1897] and *J. andesiana* [Berland, 1913]) shows relative uniformity in the femoral apophysis and high variability in the shapes of the embolus, median apophysis, paramedian apophysis and conductor (Figure 3).

### 3.2 Mating process and genital mechanics

Out of 24 mating trials, one couple of *Josa* sp. and four of *J. calilegua* where cryofixed with variable degrees of success. Only in the *Josa* sp. couple, all of the genital structures remained in functional contact, but from the relative position of the male genital structures and their proximity with the female ones in the remaining couples it can be assumed that both species use the same genital coupling mechanism (3D interactive models of the genital coupling of both studied *Josa* species are provided as supplemental files: Figures S1 and S2).

**FIGURE 4:**
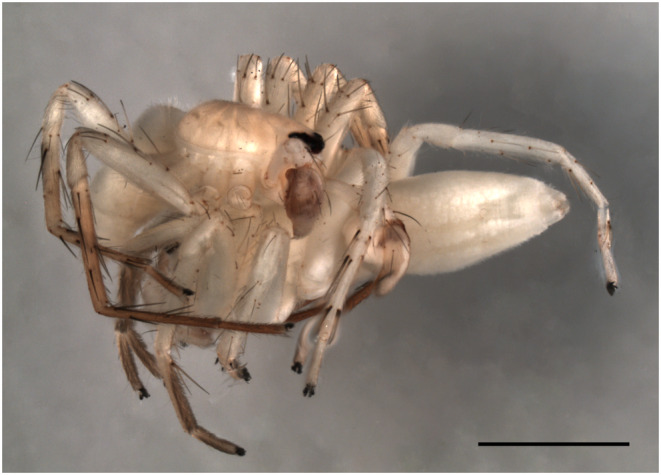
Dorsal view of cryofixed mating pair of *Josa calilegua* (DPO‐0270). Note the typical mating position of RTA clade spiders, with the male on top of the female, facing the opposite direction. Scale: 2 mm.

In all the cases mating occurred after a frontal approach of the male, with both spiders facing each other. Both spiders then briefly tapped each other with their two first pairs of legs, and then the male climbed onto the female and adopted the typical mating position of the RTA clade (von Helversen, 1976), with both spiders facing opposite directions, and the male prosoma on top of the female posterior end of the prosoma (Figure 4).

**FIGURE 5:**
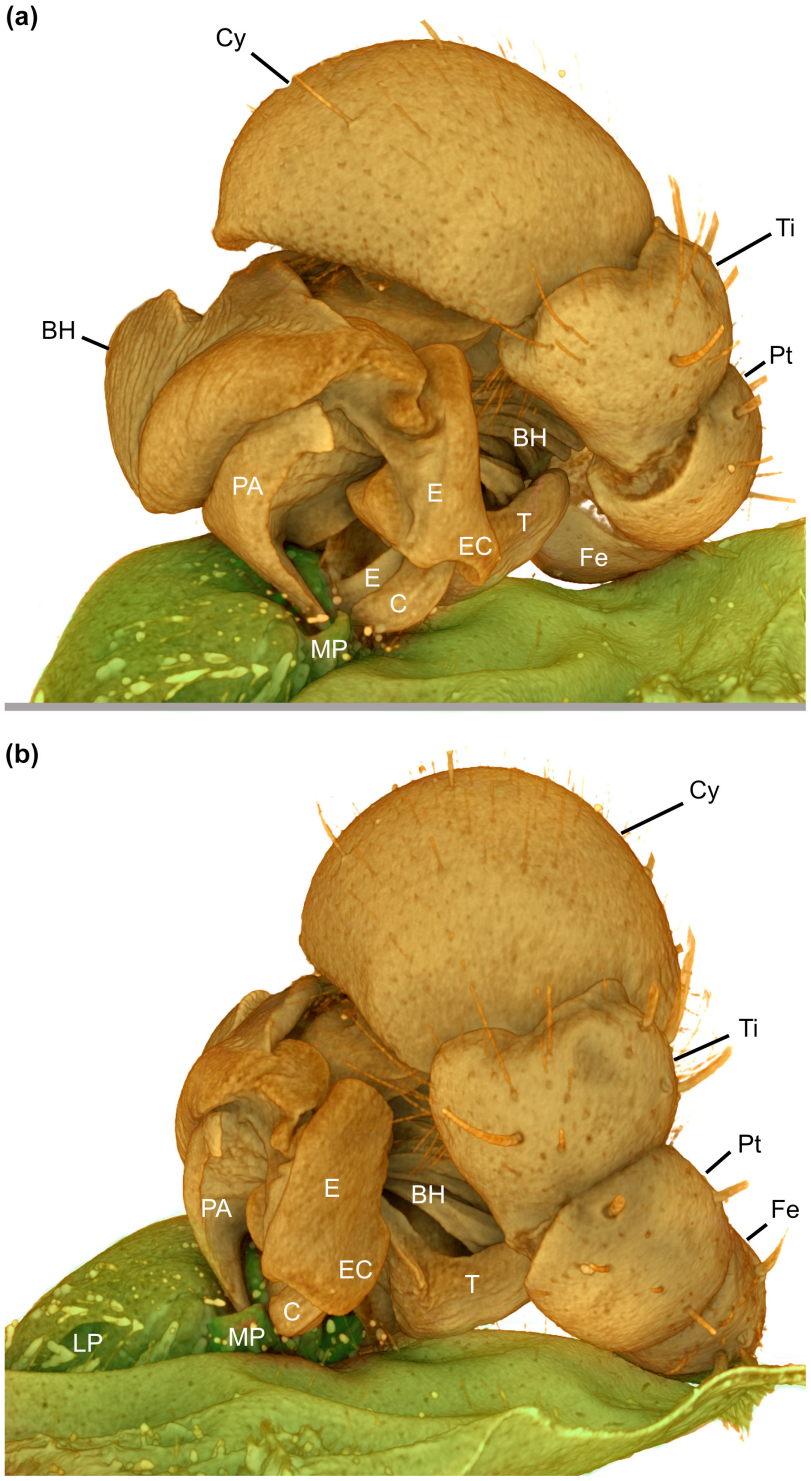
Volume rendering of lateral (a) and posterior (b) view of coupled genitalia of *Josa* sp. (MJR‐2552). BH, basal hematodocha; C, conductor; Cy, cymbium; E, embolus; EC, basal embolar crest; Fe, femur; LP, lateral pocket; MP, median pocket; PA, paramedian apophysis; Pt, patella; T, tegulum; Ti, tibia.

**FIGURE 6:**
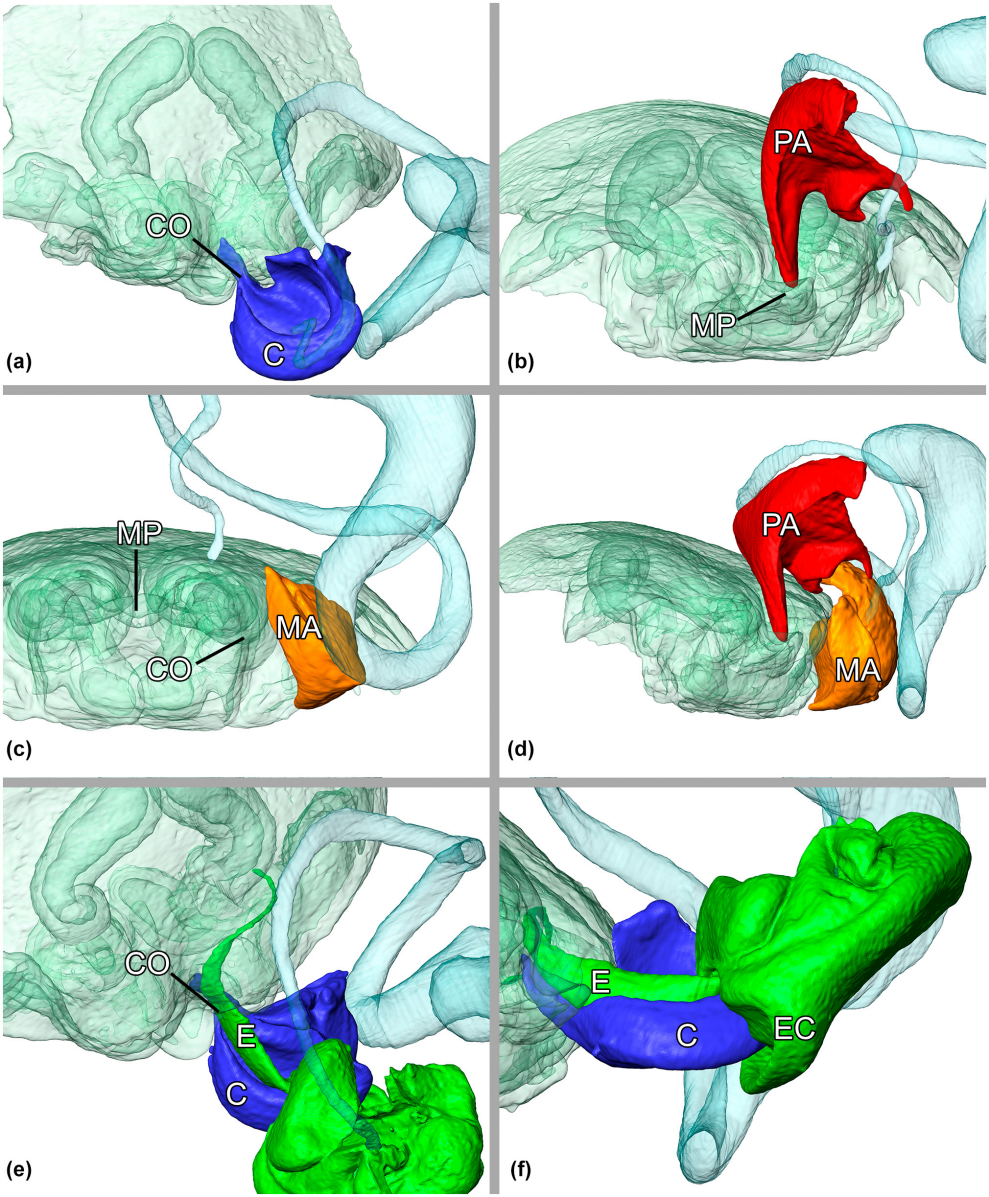
*Josa* sp. (MJR‐2552) genital mechanics. Virtual reconstructions of individual genital structures during genital coupling, the female genital structures are faintly colored in green: (a) ventral view, primary locking between the conductor and the ipsilateral copulatory opening (CO); (b) posterior view, secondary locking of paramedian apophysis (PA) and median pocket (MP); (c) posterior view, secondary locking of median apophysis (MA) in the ipsilateral lateral pocket; (d) lateral view, both secondary lockings; (e) ventral view, functional conduction of the embolus through the copulatory opening (CO); (f) lateral view, internal bracing mechanism of EC and the conductor (C) base. C, conductor; CO, ipsilateral copulatory opening; E, embolus; EC, basal embolar crest; MA, median apophysis; MP, median pocket; PA, paramedian apophysis. The spermophor is included in the representations in light blue to serve as a spatial reference.

From the relative position of the genital structures shown in the micro‐CT data (Figure 5) and direct observation during the fixation of mating pairs, the following genital interactions can be deduced: the primary locking is achieved by fitting the tip of the conductor into the ipsilateral copulatory opening (i.e., the left conductor is locked into the left copulatory opening; Figure 6a). Then, the hematodochae expand and the remaining genital interactions occur. Two secondary lockings could be observed: one with the tip of the paramedian apophysis lodged into the epigynal median pocket (Figure 6b), and the other with the median apophysis firmly locked against the ipsilateral lateral pocket (i.e., the left median apophysis is locked into the left lateral pocket; Figure 6c). The forces of those secondary lockings are convergently opposed, firmly clamping the copulatory bulb to the epigyne (Figure 6d).

The conductor holds a double function, since in addition to primary locking, it also performs the functional conduction—the lamellar part of the embolus is guided through the furrow of the conductor into the ipsilateral copulatory opening (ipsilateral insertion pattern; Figure 6e).

**FIGURE 7:**
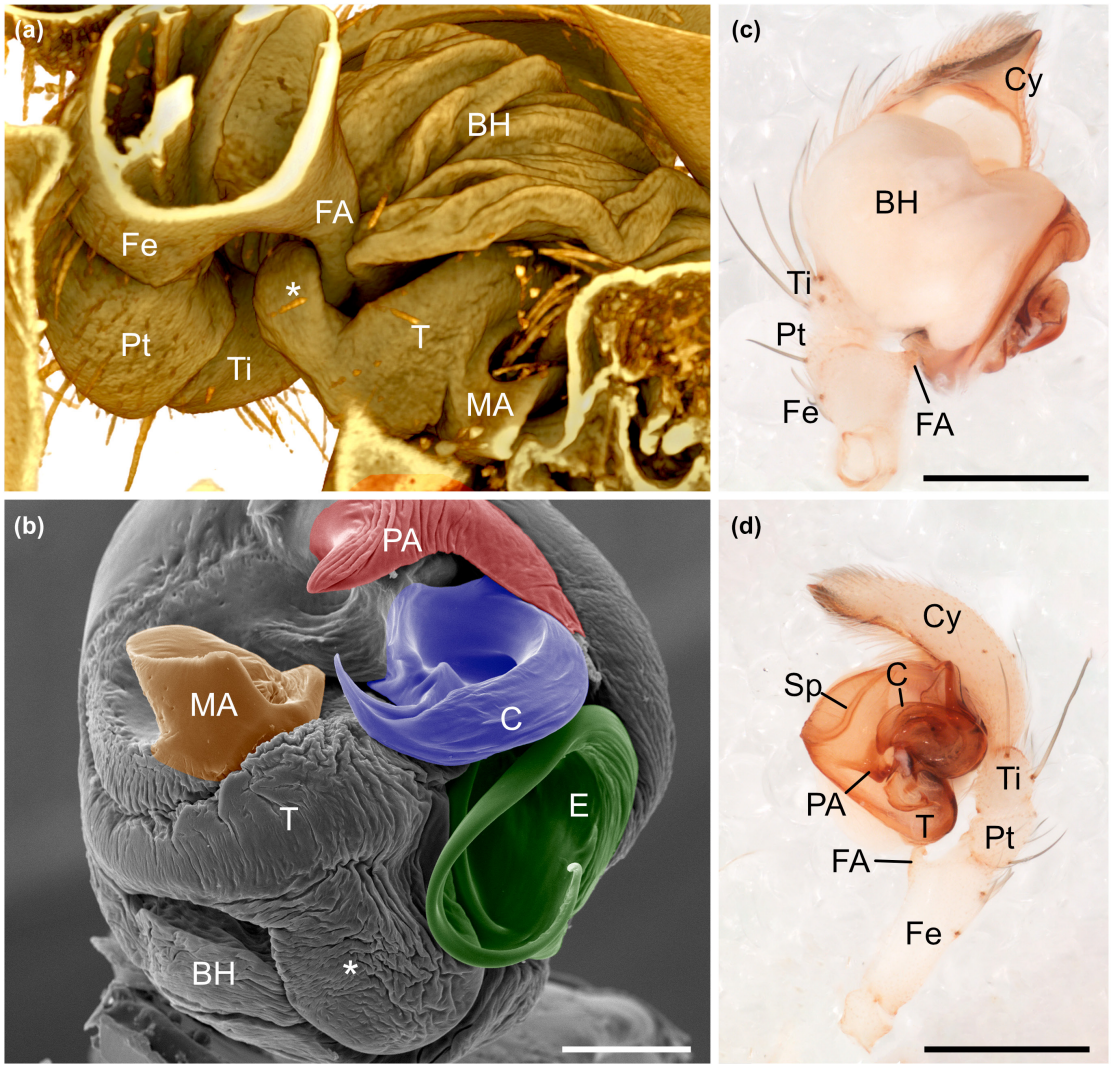
*Josa* genital mechanics. Volume rendering of the coupled genitalia of *Josa* sp. (MJR‐2552): (a) ventral view, internal bracing mechanism of femoral apophysis and tegular projection (asterisk). Dissected left copulatory bulb of *Josa* sp., SEM image: (b) apical view, note the tegular projection located dorso‐distally in the resting copulatory bulb (asterisk). Dissected left pedipalp of detached cryofixed mating pair of *Josa calilegua*: (c) ventral view; (d) retrolateral view. Note the femoral apophysis in self‐bracing position. Scales: (b) 100 μm; (c, d) 50 μm. BH, basal hematodocha; C, conductor; Cy, cymbium; E, embolus; FA, femoral apophysis; Fe, femur; MA, median apophysis; PA, paramedian apophysis; Pt, patella; Sp, spermophor; T, tegulum.

The male genitalia show two self‐bracing mechanisms. The first one occurs between the basal EC, which is locked against the base of the conductor (Figure 6f) while the lamellar part of the embolus slides through the furrow of the conductor. The second one involves the femoral apophysis and a hitherto unnoticed semisoft projection of the tegulum (Figure 7a). This tegular projection is located dorso‐distally in the resting copulatory bulb (Figure 7b), and, while expanded, it lodges against the toothed tip of the femoral apophysis. The distal denticles of the apophysis probably contribute to a strong engagement, as shown in a detached mating pair of *J. calilegua* whose male sustained the femur–bulb engagement (Figure 7c,d).

## 4 DISCUSSION

The analysis of the genital coupling of two *Josa* species clearly shows that the femoral apophysis does not contact the female genitalia, but it is instead used for self‐bracing, probably to enhance structural stability during pedipalpal insertions. Therefore, we can refute our hypothesis that the femoral apophysis is used for primary locking replacing the missing RTA.

### 4.1 Self‐bracing mechanisms

The similarity of the femoral apophysis throughout other *Josa* species suggests that its function is the same for the whole genus. This self‐bracing mechanism is interesting in several respects. The mere presence of a pedipalpal femoral apophysis is an atypical condition among spiders (Ramírez, 2014), because pedipalpal structures that engage in genitalic interactions (e.g., all the copulatory bulb sclerites, the paracymbium in Araneoidea, or the RTA) usually emerge in the most‐distal articles of the pedipalp (i.e., copulatory bulb, cymbium, and tibia), where they can easily come into contact with their functional counterparts during the genital coupling. In contrast to this usual situation in spiders, *Josa* has a particularly short patella and tibia, which seems to be crucial for the engagement of the femoral apophysis in the tegular projection, that would be otherwise too far apart to interact. A similar organization can be seen in few groups with femoral modifications: males of *Orthobula* and *Capobula* (Trachelidae) (see Haddad et al., 2022 and 2021, respectively) bear a hook‐like femoral apophysis similar to that of *Josa*, and males of *Philoponella*, *Conifaber* and *Zosis* (Uloboridae) have a basal femoral protuberance that comes into contact with the copulatory bulb in artificially expanded pedipalps (see Grismado, 2004: figs. 38 and 39). What all of these species have in common is that the pedipalps have a short patella and tibia suggesting a function in self‐bracing; this should be addressed in future studies on genital mechanics in these groups.

The presence of denticles on the tip of the femoral apophysis of *Josa* is another morphological feature that may be related to this peculiar type of self‐bracing mechanism. To our knowledge, this is the only known self‐bracing mechanism with a mostly opposing membranous structure (the tegular projection), and the denticles probably increase the friction between the femoral apophysis and the receiving projection. Thus, it can be hypothesized that hard genital structures with denticles, which are widespread in taxonomic literature (e.g., the tip of the conductor in many Amaurobioidinae, see Ramírez, 2003; the lateral apophysis of the conductor in *Diapontia*, see Piacentini et al., 2017) probably interact with soft surfaces—from a mechanical perspective, the presence of denticles can be especially beneficial when the surface of the functional counterpart is soft. Further evidence supporting this prediction is the interaction of the heavily toothed conductor and the soft copulatory opening in *Dictyna uncinata* (see Huber, 1995a: figs. 1D and 10).

It is not clear how frequently self‐bracing occurs in the RTA clade, as there are not many reliable reports of such mechanisms for the group (Huber, 1995a). However, the few studies available suggest that self‐bracing is usually achieved by interaction of tibial elements with the copulatory bulb. For example, in the Agelenidae, including *Allagelena graciliens* (Osterloh, 1922), *Agelenopsis aperta*, *A. oklhahoma* and *A. pennsylvanica* (Gering, 1953), the RTA is either used to support the median apophysis while in functional contact with the epigyne (in *A. graciliens*, see Osterloh, 1922: fig. 38), or to limit the cymbial flexure (in the *Agelenopsis* species, see Gering, 1953). In Thomisidae, studies on *Ebrechtella tricuspidata*, *Heriaeus graminicola* and *Misumena vatia* have shown that the characteristic ventral tibial apophysis (VTA) of many Thomisidae braces against the tegulum during the expansion of the copulatory bulb (Huber, 1995a; Loerbroks, 1983, 1984); instead, in *Philodromus aureolus* (Philodromidae), a similar VTA interacts with the conductor (see Huber, 1995a: fig. 12). A peculiar situation was described for the *Aysha prosepera* group (Anyphaenidae), which also have two self‐bracing mechanisms (Poy et al., 2023)—a basal tibial process that supports the base of a tibial conductor and a prolateral tibial apophysis that limits the cymbial flexure (see Poy et al., 2023: fig. 5).

Self‐bracing is also known for other spider groups. For example, it appears to be widespread in Araneoidea and is also known for some Synspermiata taxa. In araneoids, self‐bracing almost exclusively involves paracymbial modifications, such as the paracymbium in Araneidae, Linyphiidae and Tetragnathidae (Barrantes et al., 2013; Blest & Pomeroy, 1978; Eberhard & Huber, 1998; Grasshoff, 1973a, 1973b; Uhl et al., 2007; van Helsdingen, 1965, 1969), or the cymbial hood in some Theridiidae (Agnarsson et al., 2007; Knoflach, 1998). In Synspermiata, self‐bracing is known for Pholcidae, where the male pedipalps are drastically rotated prior to insertions (Huber & Eberhard, 1997), and locked in position by the bracing of a pedipalpal trochanter apophysis and a cheliceral apophysis (Huber, 1996). Interestingly, some Pholcidae have acquired independently a self‐bracing mechanism that is remarkably similar to that of *Josa*: the males of *Anopsicus zeteki*, *Psilochorus simoni* and several *Modisimus* species also lock a femoral apophysis into an indentation of the copulatory bulb during pedipalpal insertions (as in *M. culicinus*; see Huber, 1998: fig. 6).

### 4.2 Primary locking

The primary locking in the studied *Josa* species is achieved by the conductor, a structure of the copulatory bulb, fitting the ipsilateral copulatory opening of the female. This situation is comparable to the lycosid *Agalenocosa pirity*, which also lacks an RTA, like all the Lycosidae, but has reacquired a tibial apophysis in a more ventral position (Piacentini, 2014). In that case, males also rely on a structure of the copulatory bulb for primary locking (see Poy et al., 2020), although the function of the tibial apophysis is still unknown. Thus, it can be concluded that in taxa that have lost the RTA, primary locking is achieved by elements of the copulatory bulb, even when pedipalpal tibial apophyses have been re‐acquired.

### 4.3 Contact structures and the evolution of complex male genitalia

The fact that male genitalia offer a large number of characters for species delimitation is widely explained by rapid evolution due to intense sexual selection (Arnqvist, 1998; Eberhard, 1985; Simmons, 2014). The male genital organs are often complex and hold functions beyond basic sperm transfer. For example, structures that act as claspers (as in water striders, see Ronkainen et al., 2005), sperm removal devices (as in many dragonflies, see Córdoba‐Aguilar et al., 2003) and titilators (as in bushcrickets, see Wulff et al., 2015) can be found in male genital organs. The case of *Josa* fits the pattern of most spider groups, whose species can be diagnosed on the basis of male genital morphological variation (see Ramírez, 2003). It is remarkable, though, that the delimitation of species in *Josa* relies on structures that contact the female genitalia during genital coupling (i.e., the conductor, median apophysis, paramedian apophysis and embolus), and not on the femoral apophysis, a structure that is used for the structural stability of the pedipalp and is relatively uniform, compared with the remarkable variability of the genital elements that contact the female. It has been shown that sexual traits have higher rates of change than ecological or somatic traits (Gonzalez‐Voyer & Kolm, 2011; Simmons & Fitzpatrick, 2019), and our findings suggest that similar patterns of differential evolutionary dynamics may extend as well to the male genital structures. Two explanations can account for the differential morphological divergence of male genital contacting and noncontacting structures in *Josa*. The first possibility is that the male genital structures that participate in the self‐bracing mechanism are under a weaker directional selective regime than those that contact the female due to its structural role during the genital coupling, resulting in a lesser morphological divergence among species. The second possibility could be that the type, but not the intensity of the selection differs between male genital contacting and noncontacting structures. That is, the femoral apophysis of *Josa* could be under a strong stabilizing selective regime, instead of a weak directional one, which would constrain its interspecific morphological variation. Nonetheless, regardless of the different selective processes acting on male genitalia, the differential variability of male genital structures suggests that the evolution of the femoral apophysis of *Josa* is decoupled from that of the copulatory bulb sclerites, or more specifically, that there is a modularity in the evolution of the male pedipalpal structures. Therefore, *Josa* species may be a good model for studying the evolutionary dynamics of male genital structures that contact the female genitalia in contrast to those that do not.

## 5 CONCLUSIONS

We have successfully unraveled the functional role of the femoral apophysis of *Josa* and shown that it does not participate in primary locking, but is involved in self‐bracing to stabilize the male copulatory organ during copulation. Moreover, the occurrence of not one, but two self‐bracing mechanisms in *Josa* suggests that there is a selection over structural stability during pedipalpal insertions in comparison to most studied RTA clade species. This peculiarity, along with the apparent differential morphological divergence of contacting and noncontacting genital structures make *Josa* an interesting model for future behavioral and evolutionary studies.

## AUTHOR CONTRIBUTIONS

**Dante Poy:** Conceptualization (supporting); formal analysis (lead); investigation (lead); methodology (equal); writing – original draft (lead); writing – review and editing (equal). **Luis N. Piacentini:** Conceptualization (supporting); formal analysis (supporting); investigation (supporting); supervision (equal); writing – original draft (supporting); writing – review and editing (equal). **Shou‐Wang Lin:** Formal analysis (supporting); investigation (equal); methodology (equal); visualization (equal); writing – review and editing (supporting). **Leonel A. Martínez:** Formal analysis (supporting); investigation (supporting); writing – review and editing (supporting). **Martin J. Ramírez:** Conceptualization (lead); project administration (equal); resources (equal); supervision (equal); writing – original draft (equal); writing – review and editing (equal). **Peter Michalik:** Conceptualization (equal); methodology (equal); project administration (equal); resources (lead); software (lead); supervision (equal); writing – original draft (equal); writing – review and editing (equal).

## CONFLICT OF INTEREST STATEMENT

The authors declare no conflict of interest.

## Supporting information


Figure S1

Figure S2

Appendix S1

## Data Availability

The micro‐CT scans used for this study are stored in the MorphoBank (http://morphobank.org/permalink/?P4795).
