# Supplementary material for: Copulatory mechanics of ghost spiders reveals a new self‐bracing mechanism in entelegyne spiders
Source: Ecol Evol. 2023 Oct 3;13(10):e10582. doi: 10.1002/ece3.10582 (PMC10547672; doi:10.1002/ece3.10582)

Figure is best viewed with Adobe Reader 9 or later

Click inside image or frame to enable interactive mode.
- Left-click & move mouse to rotate scene.
- Right-click & move mouse to zoom.
- Both-click and move mouse to pan.

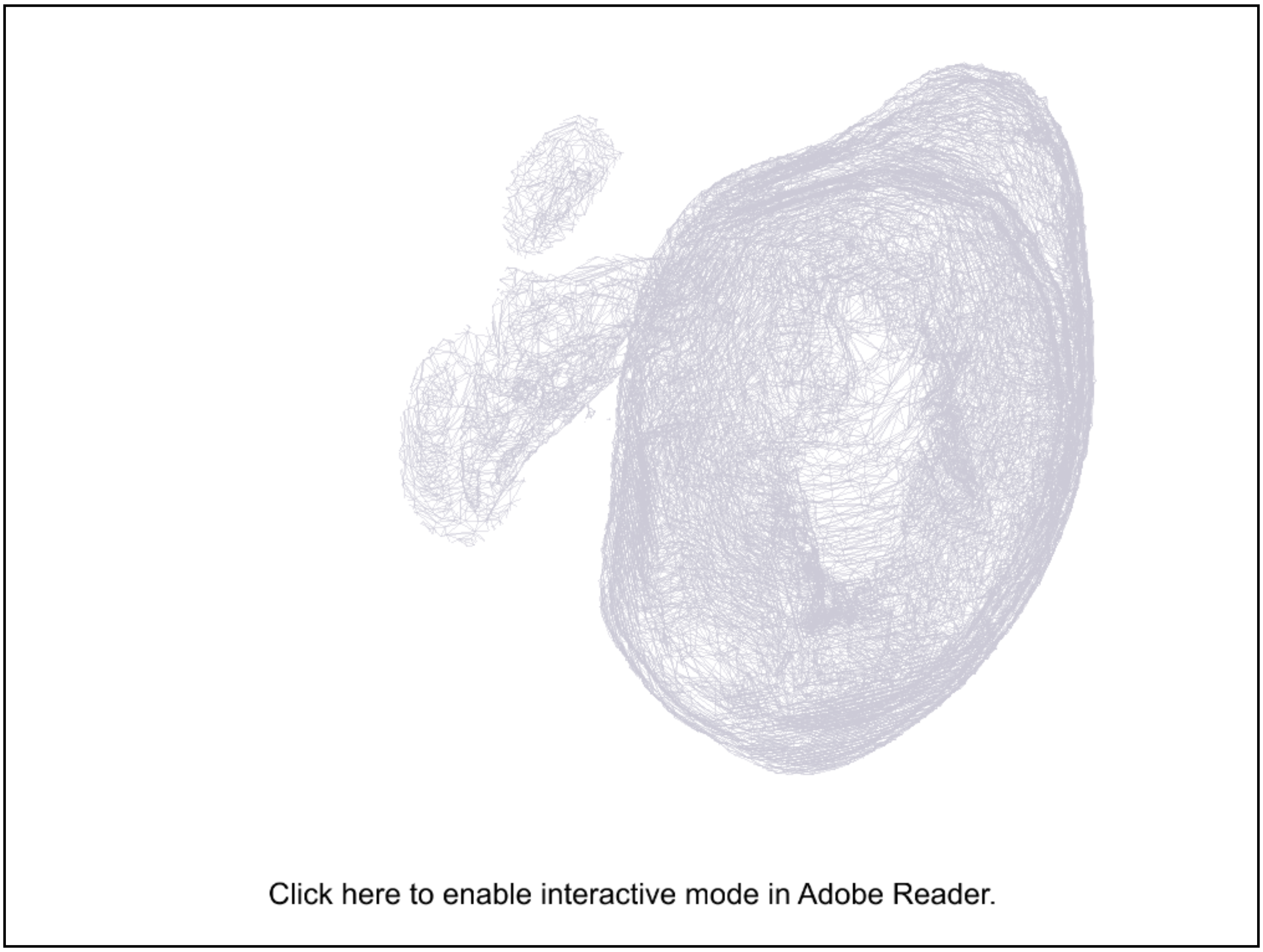

Supplement: Supplementary file 2 — Figure S2 [file ECE3-13-e10582-s002.pdf]
