# Supplementary material for: Copulatory mechanics of ghost spiders reveals a new self‐bracing mechanism in entelegyne spiders
Source: Ecol Evol. 2023 Oct 3;13(10):e10582. doi: 10.1002/ece3.10582 (PMC10547672; doi:10.1002/ece3.10582)
Supplement: Supplementary file 3 — Appendix S1 [file ECE3-13-e10582-s003.docx]

**Supplemental files**

**Supplementary figure 1**. Interactive three‐dimensional (3D) model of the coupled genitalia of *Josa* sp. (MJR-2552). The model was obtained from the cryofixed mating pair, and is included to exhibit the overall genital coupling mechanism (all genital functions are individually depicted in the figures below). The PDF version contains interactive 3D content. The colored structures correspond to the functionally relevant male structures (spermophor is colored to serve as a spatial reference). To activate, click on the figure in Adobe Reader. The model can be rotated with the mouse, and individual structures can be removed if needed; see the content menu for further functionalities. Color code: blue, conductor; green, embolus; light blue, spermophor; orange, median apophysis; pink, femoral apophysis; red, paramedian apophysis.

**Supplementary figure 2**. Interactive three‐dimensional (3D) model of the coupled genitalia of *Josa calilegua* (DPO-0270)*.* The model was obtained from the cryofixed mating pair, and is included to exhibit the overall genital coupling mechanism (all genital functions are individually depicted in the figures below). The PDF version contains interactive 3D content. The colored structures correspond to the functionally relevant male structures (spermophor is colored to serve as a spatial reference). To activate, click on the figure in Adobe Reader. The model can be rotated with the mouse, and individual structures can be removed if needed; see the content menu for further functionalities. Color code: blue, conductor; green, embolus; light blue, spermophor; orange, median apophysis; pink, femoral apophysis; red, paramedian apophysis.
